# Genetic polymorphisms of *NOS2* and predisposition to fracture non-union: A case control study based on Han Chinese population

**DOI:** 10.1371/journal.pone.0193673

**Published:** 2018-03-08

**Authors:** Wei Huang, Kun Zhang, Yangjun Zhu, Zhan Wang, Zijun Li, Jun Zhang

**Affiliations:** Department of Trauma Surgery, Honghui Hospital, Xi’an Jiaotong University Health Science Center, Xi’an, Shaanxi, China; University of Umeå, SWEDEN

## Abstract

A non-union, especially atrophic non-unions, is a permanent failure of healing following a fracture and can be difficult to treat. Approximately 5–10% of fractures will result in a non-union during the healing process. non-unions can be classified into two types: atrophic non-union which is often due to impaired bone healing with a potential biological mechanism, and hypertrophic non-union which is due to inadequate fixation after fracture. Genetic variations also play an important role in the fracture healing response. Previous studies based on animal models have indicated that *NOS2* might be greatly involved in the bone fracture healing process. In this case-control study, 346 nonunion patients were compared to 883 patients with normal fracture healing to investigate the potential genetic association between *NOS2* and the fracture healing process using study subjects of Chinese Han ancestry. Twenty-seven single nucleotide polymorphisms (SNPs) covering *NOS2* were genotyped in our study subjects and analyzed. In addition to the single marker-based analysis, we performed a gene-by-environment analysis to examine the potential interactions between genetic polymorphisms and some environmental factors. SNP rs2297514 showed significant association with the fracture healing process after adjusting for age and gender (OR = 1.38, *P* = 0.0005). Our results indicated that the T allele of rs2297514 significantly increased the risk of a non-union during the fracture healing process by 38% compared to the C allele. Further stratification analyses conducted for this SNP using data from subgroups classified by different sites of fracture indicated that significance could only be observed in the tibial diaphysis subgroup (N = 428, OR = 1.77, *P* = 0.0007) but not other groups including femur diaphysis, humeral shaft, ulnar shaft, and femur neck. Gene-by-environment interaction analyses of the three environmental factors showed no significant results. In this study, rs2297514 was significantly associated with the non-union status of fracture healing using a large Chinese population-based study sample. Our findings replicated those of a previous preliminary study and offered strong evidence linking *NOS2* and fracture healing.

## Introduction

A non-union is a permanent failure of healing following a fracture. Approximately 5–10% of fractures will result in a non-union during the healing process [1]. Non-unions, especially atrophic non-unions, can be difficult to treat. Patients with non-unions will have chronic pain at the site of the fracture and abnormal movement at the level of the fracture [1–6]. Several factors have been associated with the development of non-unions, such as smoking, the type of fracture (open or closed), and poor mechanical stability [7]. Genetic variations also play an important role in the fracture healing response [8]. With the widespread application of sequencing and genetic association analyses for studying the genetics of complex diseases, such as schizophrenia [9–12], several bone formation and growth-related genes and molecules were investigated for their potential contribution to the risk of developing a non-union during the fracture healing process, including pro-inflammatory cytokines (*IL1* and *IL6*), bone morphogenetic proteins (BMPs), and tumor necrosis factor-alpha (TNF-α) [13–15].

*NOS2* encodes the nitric oxide synthase 2 enzyme, which synthesizes nitric oxide in the human body. Nitric oxide is a reactive free radical that acts as a mediator in several biological processes, including neurotransmission and antimicrobial and antitumoral activities. In addition, it is a messenger molecule with diverse functions throughout the body. A previous study on rats has shown that an elevated expression of *NOS2* occurs during the early stages of fracture healing in endosteal osteoblasts and chondroblasts. This elevated expression peaked four days after the fracture, but the expression of *NOS2* could not be detected at four weeks post-fracture [16]. These findings indicate that *NOS2* might be highly involved in the bone fracture healing process. A preliminary population-based study by Vikram et al. examined the distribution of genetic polymorphisms in *NOS2* from non-union patients and normal fracture healing controls [15]. A potential susceptible single nucleotide polymorphism (SNP) with suggestive *P* values, rs2297514, was associated with non-unions. However, the significance of rs2297514 could not be confirmed due, at least in part, to the insufficient statistical power. In this study, we aimed to investigate the potential genetic association between *NOS2* and the fracture healing process using study subjects of Chinese Han ancestry. Twenty-seven SNPs that covered *NOS2* were genotyped in our study subjects and analyzed. In addition to the single marker-based analysis, we performed gene-by-environment analyses to examine the potential interactions between genetic polymorphisms and some environmental factors.

## Methods

### Study subjects

This hospital-based case-control study included 1,229 unrelated patients with long bone fractures who were admitted to and treated at Honghui Hospital of Xi'an Jiaotong University from 2012 to 2016. We only included Han Chinese patients who were born in the local area in an effort to have a genetically homogenous cohort of individuals. All had undergone routine post-injury check-up at least 9 months after the fractures. Non-union was defined as the cessation of all healing processes and failure to achieve union within 9 months without radiographic signs of progression of the fracture callus. Union was defined as radiographic and clinical evidence that the fracture had healed by six months and an absence of pain without movement at the fracture site or no pain with full weight bearing. Exclusion criteria included individuals with a known systemic inflammatory disease, osteoporosis and other metabolic bone diseases, pathological fractures and subsequent non-unions, hypertrophic and infected non-unions, pregnancy, and being under 18 years of age. The bone mineral density of all subjects was measured using dual-energy X-ray absorptiometry (Lunar Expert 1313, Lunar Corp., USA). Osteoporosis was diagnosed according to the criteria of the World Health Organization. None of the subjects had a history of taking medicines for the treatment of osteoporosis, and subjects with diseases or medications known to affect bone metabolism were also excluded from the study. The non-union group consisted of 346 patients (199 males and 147 females) with delayed bone healing, and the union group was composed of 883 patients (505 males and 378 females). Relevant clinical characteristics and demographics for patients were reviewed at the time that the study was conducted and are shown in Table 1. Written informed consent was obtained from each subject. The study protocol conformed to the ethical guidelines of the 1975 Declaration of Helsinki and was approved by the Ethics Committee of the Xi’an Honghui Hospital (No. 2012006).

**Table 1:** Demographic and clinical characteristics of the subjects.

Characteristics	Subjects (N = 1,229)		*P*-value
	Non-union patients (%)	Union controls (%)	
Number	346 (28.15)	883 (71.85)	-
Age, mean±SD (years)	46.12±8.09	44.74±8.27	0.0076
Male / Female	199 / 147	505 / 378	0.9690
Smoking			
Yes	44 (12.72)	125 (14.16)	
No	302 (87.28)	758 (85.84)	0.5707
Alcohol consumption			
Yes	164 (47.40)	470 (53.23)	
No	182 (52.60)	413 (46.77)	0.0758
Fracture Type			
Open	77 (22.25)	240 (27.18)	
Closed	269 (77.75)	643 (72.82)	0.0886
Fracture site			
Tibial diaphysis	113 (32.66)	315 (35.67)	
Femur diaphysis	98 (28.32)	233 (26.39)	
Humeral shaft	82 (23.70)	188 (21.29)	
Ulnar shaft	39 (11.27)	117 (13.25)	
Femur neck	14 (4.05)	30 (4.40)	0.6074

SD: standard deviation. T test was conducted for quantitative variable and chi square test was utilized for qualitative variables, such as gender, smoking status etc.

### SNP selection and experimental methods

The genetic markers utilized in this study were generated through a two-stage process. We first extracted SNPs with a minor allele frequency (MAF) greater than 0.01 based on the 1000 Genomes Project Chinese population data within the *NOS2* gene region [17]. A total of 112 SNPs were selected during this process. Then, we selected the tagging SNPs that captured most of the information offered by these 112 SNPs using the SNP-tagging algorithm Tagger [18] and an r^2^ threshold of 0.6. Finally, the number of selected SNPs was reduced to 27. The basic information from these 27 SNPs is summarized in S1 Table. These SNPs all passed the Hardy-Weinberg Equilibrium (HWE) test in our study.

Peripheral blood was drawn from a vein into a sterile tube containing ethylenediamine tetraacetic acid (EDTA). Genomic DNA was extracted from peripheral blood leukocytes according to the manufacturer's protocol (Genomic DNA kit, Axygen Scientific Inc., CA, USA). DNA was stored at -80°C for further genotyping. Genotyping was performed for all SNPs using the MassARRAY platform (Sequenom, San Diego, CA, USA). Briefly, SNPs were genotyped using high-throughput, matrix-assisted laser desorption ionization–time-of-flight (MALDI–TOF) mass spectrometry. The resulting spectra were processed using Typer Analyzer software (Sequenom, San Diego, CA, USA), and genotyping data were generated from the samples. The case/control status of the samples was concealed during the genotyping processes [19]. A randomly chosen 5% of the samples was repeated, and the results were 100% concordant [20]. The final genotyping call rate of each SNP was greater than 99%, and the overall genotyping call rate was 99.9%. The quality of our genotyping results ensured the reliability of further statistical analyses.

### Statistical and bioinformatics analysis

We performed the genetic association study by fitting logistic models. SNPs were coded as 0, 1 or 2 when there were 0, 1 or 2 minor alleles, respectively (additive model). According to Table 1, only age was significantly associated with the distribution of fracture non-union status (*P* = 0.0076). Therefore, in our logistic model, we included only age and gender as covariates. To examine the potential effects of gene-environment interactions (GxE) for our selected SNPs, we also fitted additional logistic models by adding the three environmental factors of smoking, alcohol and type of fracture as main factors and another factor, the product of each environmental factor and the SNPs. In addition, we stratified our data by the five different fracture sites (tibial diaphysis [TD], femur diaphysis [FD], humeral shaft [HS], ulnar shaft [US], and femur neck [FN]) for our significant SNPs to examine whether the site of fracture affected our genetic association signals. Bonferroni corrections were applied for multiple comparisons, and the main *P* value threshold used in this study was 0.05/27≈0.002. The statistical computing software R project [21] and genetic analysis software Plink v1.9 (http://www.cog-genomics.org/plink2/) [22] were utilized for the analyses described above.

In addition to the statistical analysis, we performed a bioinformatics analysis to investigate the potential functional significance of our targeted SNPs and the candidate genes associated with *NOS2*. Two tools were utilized to evaluate the potential biological functions of SNPs. The first one is RegulomeDB (http://www.regulomedb.org/), which assesses SNPs by integrating data from the ENCODE project [23]. The other tool is the GTEx database (https://gtexportal.org/home/), which evaluates the potential functional significance of SNPs by exploring their effects on gene expression in multiple human tissues [24]. In addition, we examined the subnetwork around our candidate gene *NOS2* using the protein-protein interaction (PPI) database STRING (https://string-db.org/) [25].

## Results

SNP rs2297514 was significantly associated with the fracture healing process after adjusting for age and gender (OR = 1.38, *P* = 0.0005). Our results indicated that the T allele of rs2297514 significantly increased the risk of non-union during the fracture healing process by 38% compared to the C allele (Table 2). Further stratification analyses conducted for this SNP using data from different sites of fracture subgroups indicated that significance was only observed in the TD subgroup (N = 428, OR = 1.77, *P* = 0.0007), and no significant results were observed from the other four subgroups (Table 3). Since TD is the largest of these five subgroups, to ensure that this difference in significance was not a result of the insufficient statistical power caused by a small sample size, we also conducted an analysis using the combined data from the other four subgroups (FD+HS+US+FN). Again, the result was not significant (N = 801, OR = 1.24, *P* = 0.0564). Our results indicated that there is a site of fracture-specific pattern for the genetic effects of SNP rs2297514 on the fracture healing process. Gene-by-environment interaction analyses of the three environmental factors showed no significance (S2 Table). Two SNPs (rs28999409 and rs28942370) showed nominal significance for the statistical interaction between type of fracture and genetic markers; however, the significance levels were not sufficient to claim positive hits due to multiple comparisons.

**Table 2:** Single marker based association analysis using logistic model adjusted by age and gender.

CHR	SNP	BP	A1	OR	STAT	*P*
17	rs28944211	27757887	A	1.01	0.06	0.9525
17	rs28944196	27762201	G	1.04	0.39	0.6970
17	rs28944186	27763200	G	0.98	-0.15	0.8781
17	rs2297514	27766289	T	1.38	3.49	0.0005
17	rs149411888	27769376	C	0.94	-0.30	0.7612
17	rs28999412	27770967	T	0.94	-0.34	0.7367
17	rs28999409	27771612	A	0.97	-0.28	0.7781
17	rs28999406	27772735	A	1.12	0.44	0.6621
17	rs2248814	27773295	A	0.82	-2.02	0.0431
17	rs118160614	27773934	T	0.96	-0.19	0.8485
17	rs142205241	27774560	C	0.93	-0.38	0.7070
17	rs144645983	27777837	C	1.02	0.07	0.9441
17	rs28999380	27778549	G	0.86	-0.79	0.4324
17	rs944724	27782391	T	0.90	-1.00	0.3189
17	rs56114296	27783722	A	0.93	-0.42	0.6740
17	rs3794761	27784170	A	1.04	0.38	0.7003
17	rs28942370	27787362	G	0.90	-0.71	0.4771
17	rs28730832	27788830	A	0.96	-0.21	0.8299
17	rs28998828	27790579	T	0.96	-0.35	0.7294
17	rs28998826	27791070	A	1.00	-0.02	0.9829
17	rs12452167	27794716	G	1.09	0.48	0.6297
17	rs3794766	27794895	T	0.96	-0.37	0.7149
17	rs28998814	27795159	A	1.03	0.19	0.8475
17	rs3730013	27798892	A	0.97	-0.28	0.7793
17	rs28998800	27799060	C	0.93	-0.33	0.7410
17	rs28998798	27799131	G	1.08	0.44	0.6579
17	rs6505483	27799319	A	0.98	-0.22	0.8292

**Table 3:** Stratification analysis based on the site of fracture for genetic association of rs2297514.

Site of Fracture	Sample Size		OR	STATS	*P*
	Non-Union	Normal			
Tibial Diaphysis (TD)	113	315	1.77	3.41	0.0007
Femur Diaphysis (FD)	98	233	1.41	2.01	0.0443
Humeral Shaft (HS)	82	188	1.34	1.57	0.1175
Ulnar Shaft (US)	39	117	0.97	-0.12	0.9036
Femur Neck (FN)	14	30	0.68	-0.91	0.3625
Combined without TD	233	568	1.24	1.91	0.0564

RegulomeDB scores for the 27 selected SNPs are summarized in S3 Table. One SNP, rs3794766, had a score of 1b, which indicated that it had a significant biological function. However, this SNP was not significantly associated with the fracture healing process (OR = 0.96, *P* = 0.7149). In contrast, SNP rs2297514, the significant SNP identified by association analysis, had a score of 7, which indicated that this SNP had no functional significance. We examined the gene expression patterns of rs2297514 in 40 human tissues (S4 Table). The most significant tissue was spleen (effect size = 0.47, *P* = 0.0025). However, due to multiple comparisons, this finding was not significant. Several related genes, including *UBC*, *SPSB2*, *SPSB1*, *MT-CO2*, *MAPK14*, and *IL12B*, were identified as highly connected to our target gene *NOS2* in the human PPI network (S1 Fig).

## Discussion

Preliminary studies based on European populations have identified rs2297514 in *NOS2* as a suggestive susceptible polymorphism that contributes to the risk of non-union during the fracture healing process (OR = 3.98, *P* = 0.015) [15]. In our study, we recruited more than 1,300 study subjects and selected 27 candidate SNPs within the *NOS2* gene region to ensure better coverage. Our findings replicated and validated Vikram et al.'s study and can thus be considered strong evidence for the potential genetic association between *NOS2* and the fracture healing process. Additionally, we included environmental factors such as smoking status and alcohol use and investigated the potential gene-by-environment interactions between those selected SNPs and environmental factors. In this sense, our research is more than just a replication study of the previous preliminary study.

The results of the stratification analysis by the site of fracture for our study subjects were particularly interesting. None of the previous genetic association studies focusing on non-unions have classified their study subjects by the site of fracture [14,15]. Our study found that for fractures of the tibial diaphysis, SNP rs2297514 was significantly associated with the fracture healing process. However, this association signal was not identified for the other four fracture sites. The insufficient statistical power due to a smaller sample size caused by stratification might partly explain this, especially when we consider that the TD subgroup was the largest of the five subgroups. However, when we combined the remaining four subgroups (the sample size of the merged subgroup was approximately double the sample size of TD), this new larger subgroup did not provide significant results. If we carefully examine the results of stratification analysis, an interesting pattern was observed: significant or suggestively significant association signals of rs2297514 were identified only in bones of the lower limbs (*P*_TD_ = 0.0007 and *P*_FD_ = 0.0443). For fractures in the bones of the upper limbs (HS and US), no significant signals were observed.

In this study, we also included three environmental factors to investigate their potential effects and their interaction with *NOS2*. However, none of these analyses resulted in significant hits (Table 1 and S2 Table). Among these environmental factors, smoking status has previously been reported to be related to fracture healing and non-union status. However, in our study, we failed to replicate these findings. In addition, the interaction analysis between the significant SNP rs2297514 and these three environmental factors failed to identify any significant findings. A potential reason for this is the limitation of our sample size. Although, our study had a much larger sample size compared to previous studies, it might be still not large enough to provide sufficient statistical power for detecting gene-by-environment interactions, which require larger sample sizes compared with single marker-based association analyses to achieve satisfactory statistical power.

Our bioinformatics analysis using RegulomeDB and GTEx did not identify any clues for the functional significance of SNP rs2297514. This SNP is not located in either an exonic region involved in protein coding or a regulatory region that could be involved in regulating gene expression. Combining all of the evidence, this SNP might only serve as a surrogate of some underlying susceptible variants. The underlying variant(s) were definitely not genotyped in our study and could be either another common SNP or a set of rare or low-frequency variants. In this sense, sequencing-based replication studies are needed to unravel these susceptible variants in the future. Several genes were identified as connected to *NOS2* (S1 Fig) based on a PPI network using STRING. Most of these genes were related to the human immune system, which plays an important role in the fracture healing process, and future studies focusing on these genes may be needed.

Several limitations in study design might hinder the credibility of this study. First, in this study, we examined only SNPs located within the *NOS2* gene region. Early research has shown that regions 20 kb up- and downstream of genes might contain some important regulatory elements and that variants within these regions could significantly affect gene expression levels [26–28]. Given of the limitations of single SNP analyses [29–35], for future replication studies, it is necessary to conduct haplotype analyses to provide further statistical evidence for our findings, and it is also important to consider those regions and investigate their potential contributions to the risk of non-union in the fracture healing process thoroughly. Additionally, as a candidate gene-based association study, we cannot systematically control the potential population stratification by some of the standard methods applied in GWAS. Therefore, the significant findings of our study may be false positives, although this chance is very low. During the study subject recruitment process, we restricted the genetic background of our samples by only including those who were born in the local area. This procedure may, at least partly, address this issue. We have included neck of femur fractures in our study subjects which might introduce some heterogeneity to our study results. In addition, as a single hospital based study, our study results can be very regional specific and therefore in future, replication studies based on some other populations are necessary. Last but not least, in this study we failed to consider the potential confounding effects of Vitamin D and use of NSAIDs during the period of fracture healing, and therefore we need to be careful when explaining our significant findings.

## Conclusions

In this study, we found that rs2297514 was significantly associated with the non-union status of fracture healing in a large Chinese population-based study sample. Our findings replicated those of a previous preliminary study and offered strong evidence linking *NOS2* and fracture healing. Follow-up studies using sequencing to examine the effects of rare and low-frequency variants and larger sample sizes to investigate the potential gene-by-environment interactions will be needed in the future.

## Supporting information

S1 Table Basic information of the 27 selected SNPs. (DOCX)

S2 Table Gene by environmental interactions between 27 SNPs and three environmental factors. (DOCX)

S3 Table Regulome DB score for all of these 27 selected SNPs. (DOCX)

S4 Table Tissues specific eQTL pattern of SNP rs2297514 based on data from 40 human tissues. (DOCX)

S1 Fig Gene network of NOS2 constructed by protein-protein interaction (PPI) data. (DOCX)
