# Supplementary material for: Genetic polymorphisms of NOS2 and predisposition to fracture non-union: A case control study based on Han Chinese population
Source: PLoS One. 2018 Mar 8;13(3):e0193673. doi: 10.1371/journal.pone.0193673 (PMC5843262; doi:10.1371/journal.pone.0193673)
Supplement: S1 Table — (DOCX) [file pone.0193673.s001.docx]

Supplemental Table S1. Basic information of the 27 selected SNPs

| CHR | SNP | POS | ALLELE | FUNC | GENE | MAF | HWE |
| --- | --- | --- | --- | --- | --- | --- | --- |
| 17 | rs28944211 | 27757887 | C/T | intron | *NOS2* | 0.04 | 0.3456 |
| 17 | rs28944196 | 27762201 | C/T | intron | *NOS2* | 0.34 | 1.0000 |
| 17 | rs28944186 | 27763200 | C/T | intron | *NOS2* | 0.10 | 0.5880 |
| 17 | rs2297514 | 27766289 | C/T | intron | *NOS2* | 0.43 | 0.4032 |
| 17 | rs149411888 | 27769376 | C/T | intron | *NOS2* | 0.05 | 0.4417 |
| 17 | rs28999412 | 27770967 | A/G | coding-synon | *NOS2* | 0.05 | 0.3377 |
| 17 | rs28999409 | 27771612 | C/T | intron | *NOS2* | 0.16 | 0.5275 |
| 17 | rs28999406 | 27772735 | C/T | intron | *NOS2* | 0.03 | 0.1505 |
| 17 | rs2248814 | 27773295 | A/G | intron | *NOS2* | 0.34 | 0.3037 |
| 17 | rs118160614 | 27773934 | A/T | intron | *NOS2* | 0.06 | 0.2054 |
| 17 | rs142205241 | 27774560 | C/T | intron | *NOS2* | 0.05 | 0.5001 |
| 17 | rs144645983 | 27777837 | C/G | intron | *NOS2* | 0.04 | 0.2273 |
| 17 | rs28999380 | 27778549 | C/G | intron | *NOS2* | 0.06 | 0.3726 |
| 17 | rs944724 | 27782391 | C/T | intron | *NOS2* | 0.25 | 0.9301 |
| 17 | rs56114296 | 27783722 | A/G | intron | *NOS2* | 0.07 | 0.5835 |
| 17 | rs3794761 | 27784170 | A/C | intron | *NOS2* | 0.31 | 0.9372 |
| 17 | rs28942370 | 27787362 | A/C | intron | *NOS2* | 0.10 | 0.4692 |
| 17 | rs28730832 | 27788830 | A/T | coding-synon | *NOS2* | 0.05 | 0.3377 |
| 17 | rs28998828 | 27790579 | A/G | intron | *NOS2* | 0.15 | 0.8945 |
| 17 | rs28998826 | 27791070 | C/T | intron | *NOS2* | 0.05 | 0.4417 |
| 17 | rs12452167 | 27794716 | A/G | intron | *NOS2* | 0.06 | 0.1367 |
| 17 | rs3794766 | 27794895 | C/T | intron | *NOS2* | 0.19 | 0.8261 |
| 17 | rs28998814 | 27795159 | C/T | intron | *NOS2* | 0.08 | 0.3827 |
| 17 | rs3730013 | 27798892 | C/T | intron | *NOS2* | 0.33 | 0.6470 |
| 17 | rs28998800 | 27799060 | A/G | intron | *NOS2* | 0.05 | 0.4417 |
| 17 | rs28998798 | 27799131 | C/T | intron | *NOS2* | 0.06 | 0.2199 |
| 17 | rs6505483 | 27799319 | A/G | intron | *NOS2* | 0.45 | 0.7862 |

CHR: chromosome. POS: Genomic coordinates of SNP. FUNC: function. MAF: minor allele frequency. HWE: Hardy-Weinberg Equilibrium.
