# Supplementary material for: Genetic polymorphisms of NOS2 and predisposition to fracture non-union: A case control study based on Han Chinese population
Source: PLoS One. 2018 Mar 8;13(3):e0193673. doi: 10.1371/journal.pone.0193673 (PMC5843262; doi:10.1371/journal.pone.0193673)
Supplement: S2 Table — (DOCX) [file pone.0193673.s002.docx]

Supplemental table S2. Gene by environmental interactions between 27 SNPs and three environmental factors.

| CHR | SNP | BP | A1 | Smoking | | | | Alcohol | | | | Type of Fracture | | | |
| --- | --- | --- | --- | --- | --- | --- | --- | --- | --- | --- | --- | --- | --- | --- | --- |
|  |  |  |  | OR | SE | Z | *P* | OR | SE | Z | *P* | OR | SE | Z | *P* |
| 17 | rs28944211 | 27757887 | A | 0.83 | 0.73 | -0.25 | 0.8027 | 1.42 | 0.46 | 0.76 | 0.4457 | 2.14 | 0.56 | 1.36 | 0.1731 |
| 17 | rs28944196 | 27762201 | G | 1.14 | 0.28 | 0.45 | 0.6526 | 1.25 | 0.19 | 1.16 | 0.2476 | 0.70 | 0.23 | -1.56 | 0.1186 |
| 17 | rs28944186 | 27763200 | G | 1.26 | 0.40 | 0.58 | 0.5596 | 0.98 | 0.30 | -0.08 | 0.9343 | 0.89 | 0.36 | -0.33 | 0.7383 |
| 17 | rs2297514 | 27766289 | T | 1.34 | 0.29 | 1.00 | 0.3171 | 1.01 | 0.18 | 0.08 | 0.9389 | 1.20 | 0.22 | 0.81 | 0.4155 |
| 17 | rs149411888 | 27769376 | C | 0.60 | 0.78 | -0.66 | 0.5074 | 1.40 | 0.42 | 0.80 | 0.4264 | 1.24 | 0.55 | 0.39 | 0.6930 |
| 17 | rs28999412 | 27770967 | T | 0.72 | 0.84 | -0.39 | 0.7000 | 1.36 | 0.40 | 0.78 | 0.4337 | 1.41 | 0.43 | 0.81 | 0.4195 |
| 17 | rs28999409 | 27771612 | A | 1.97 | 0.37 | 1.83 | 0.0665 | 1.11 | 0.25 | 0.41 | 0.6822 | 0.49 | 0.33 | -2.17 | 0.0303 |
| 17 | rs28999406 | 27772735 | A | 0.37 | 1.13 | -0.87 | 0.3840 | 0.70 | 0.52 | -0.68 | 0.4978 | 1.25 | 0.70 | 0.32 | 0.7494 |
| 17 | rs2248814 | 27773295 | A | 0.87 | 0.30 | -0.48 | 0.6324 | 0.81 | 0.19 | -1.09 | 0.2759 | 1.02 | 0.23 | 0.07 | 0.9403 |
| 17 | rs118160614 | 27773934 | T | 0.49 | 0.65 | -1.08 | 0.2786 | 1.19 | 0.39 | 0.45 | 0.6551 | 1.60 | 0.47 | 1.01 | 0.3108 |
| 17 | rs142205241 | 27774560 | C | 0.88 | 0.64 | -0.20 | 0.8407 | 0.67 | 0.42 | -0.96 | 0.3378 | 2.70 | 0.46 | 2.16 | 0.0306 |
| 17 | rs144645983 | 27777837 | C | 0.19 | 1.08 | -1.53 | 0.1256 | 0.99 | 0.43 | -0.03 | 0.9741 | 1.34 | 0.47 | 0.63 | 0.5280 |
| 17 | rs28999380 | 27778549 | G | 0.20 | 1.05 | -1.52 | 0.1295 | 1.09 | 0.38 | 0.23 | 0.8200 | 1.92 | 0.43 | 1.52 | 0.1286 |
| 17 | rs944724 | 27782391 | T | 0.79 | 0.34 | -0.69 | 0.4889 | 0.72 | 0.21 | -1.58 | 0.1140 | 1.57 | 0.25 | 1.81 | 0.0698 |
| 17 | rs56114296 | 27783722 | A | 0.95 | 0.53 | -0.11 | 0.9146 | 1.03 | 0.36 | 0.07 | 0.9449 | 1.61 | 0.40 | 1.18 | 0.2390 |
| 17 | rs3794761 | 27784170 | A | 1.20 | 0.27 | 0.66 | 0.5073 | 1.15 | 0.19 | 0.70 | 0.4814 | 1.59 | 0.24 | 1.96 | 0.0502 |
| 17 | rs28942370 | 27787362 | G | 1.41 | 0.45 | 0.77 | 0.4387 | 1.02 | 0.31 | 0.07 | 0.9470 | 2.35 | 0.35 | 2.43 | 0.0149 |
| 17 | rs28730832 | 27788830 | A | 2.32 | 0.52 | 1.62 | 0.1047 | 1.01 | 0.40 | 0.03 | 0.9749 | 1.97 | 0.47 | 1.44 | 0.1502 |
| 17 | rs28998828 | 27790579 | T | 0.59 | 0.41 | -1.29 | 0.1973 | 0.83 | 0.26 | -0.73 | 0.4665 | 0.79 | 0.31 | -0.76 | 0.4469 |
| 17 | rs28998826 | 27791070 | A | 0.49 | 0.82 | -0.87 | 0.3827 | 1.20 | 0.42 | 0.43 | 0.6657 | 0.81 | 0.50 | -0.43 | 0.6640 |
| 17 | rs12452167 | 27794716 | G | 0.85 | 0.64 | -0.26 | 0.7964 | 1.46 | 0.36 | 1.04 | 0.2965 | 0.50 | 0.44 | -1.57 | 0.1161 |
| 17 | rs3794766 | 27794895 | T | 0.83 | 0.39 | -0.46 | 0.6456 | 1.12 | 0.23 | 0.48 | 0.6284 | 0.76 | 0.28 | -0.98 | 0.3295 |
| 17 | rs28998814 | 27795159 | A | 0.38 | 0.67 | -1.46 | 0.1430 | 1.06 | 0.32 | 0.17 | 0.8676 | 0.61 | 0.42 | -1.19 | 0.2346 |
| 17 | rs3730013 | 27798892 | A | 0.90 | 0.28 | -0.36 | 0.7207 | 0.66 | 0.19 | -2.13 | 0.0328 | 0.66 | 0.24 | -1.74 | 0.0816 |
| 17 | rs28998800 | 27799060 | C | 0.29 | 0.79 | -1.58 | 0.1132 | 0.78 | 0.43 | -0.58 | 0.5632 | 0.32 | 0.65 | -1.77 | 0.0770 |
| 17 | rs28998798 | 27799131 | G | 0.43 | 0.60 | -1.40 | 0.1620 | 1.08 | 0.36 | 0.22 | 0.8249 | 0.35 | 0.52 | -2.03 | 0.0422 |
| 17 | rs6505483 | 27799319 | A | 0.81 | 0.28 | -0.75 | 0.4541 | 1.18 | 0.18 | 0.93 | 0.3531 | 0.87 | 0.22 | -0.63 | 0.5296 |
