# Supplementary material for: Genetic polymorphisms of NOS2 and predisposition to fracture non-union: A case control study based on Han Chinese population
Source: PLoS One. 2018 Mar 8;13(3):e0193673. doi: 10.1371/journal.pone.0193673 (PMC5843262; doi:10.1371/journal.pone.0193673)
Supplement: S3 Table — (DOCX) [file pone.0193673.s003.docx]

Supplemental table S3. Regulome DB score for all of these 27 selected SNPs.

| CHR | SNP | BP | A1 | SCORE |
| --- | --- | --- | --- | --- |
| 17 | rs28944211 | 27757887 | A | 5 |
| 17 | rs28944196 | 27762201 | G | 5 |
| 17 | rs28944186 | 27763200 | G | 3a |
| 17 | rs2297514 | 27766289 | T | 7 |
| 17 | rs149411888 | 27769376 | C | 5 |
| 17 | rs28999412 | 27770967 | T | 5 |
| 17 | rs28999409 | 27771612 | A | 5 |
| 17 | rs28999406 | 27772735 | A | 5 |
| 17 | rs2248814 | 27773295 | A | 5 |
| 17 | rs118160614 | 27773934 | T | 5 |
| 17 | rs142205241 | 27774560 | C | 5 |
| 17 | rs144645983 | 27777837 | C | 7 |
| 17 | rs28999380 | 27778549 | G | 4 |
| 17 | rs944724 | 27782391 | T | 5 |
| 17 | rs56114296 | 27783722 | A | 5 |
| 17 | rs3794761 | 27784170 | A | 5 |
| 17 | rs28942370 | 27787362 | G | 6 |
| 17 | rs28730832 | 27788830 | A | 5 |
| 17 | rs28998828 | 27790579 | T | 5 |
| 17 | rs28998826 | 27791070 | A | 5 |
| 17 | rs12452167 | 27794716 | G | 4 |
| 17 | rs3794766 | 27794895 | T | 1b |
| 17 | rs28998814 | 27795159 | A | 4 |
| 17 | rs3730013 | 27798892 | A | 5 |
| 17 | rs28998800 | 27799060 | C | 5 |
| 17 | rs28998798 | 27799131 | G | 5 |
| 17 | rs6505483 | 27799319 | A | 5 |

SCORE: Functional significance score extracted from RegulomeDB. The score system used in RegulomeDB indicates the potential biological function of a specific SNP. The range of the score is from 1-7. A smaller RegulomeDB score indicates that there are more evidence indicating biological functional significance for this specific SNP.
