# Supplementary material for: Genetic polymorphisms of NOS2 and predisposition to fracture non-union: A case control study based on Han Chinese population
Source: PLoS One. 2018 Mar 8;13(3):e0193673. doi: 10.1371/journal.pone.0193673 (PMC5843262; doi:10.1371/journal.pone.0193673)
Supplement: S4 Table — (DOCX) [file pone.0193673.s004.docx]

Supplemental table S4. Tissues specific eQTL pattern of SNP rs2297514 based on data from 40 human tissues.

| Tissue | Effect Size | T-Statistic | Standard Error | *P*-Value |
| --- | --- | --- | --- | --- |
| Spleen | 0.47 | 3.10 | 0.15 | 0.0025 |
| Esophagus - Gastroesophageal Junction | 0.33 | 3.00 | 0.11 | 0.0031 |
| Adipose - Subcutaneous | 0.16 | 2.30 | 0.07 | 0.0210 |
| Uterus | 0.27 | 2.20 | 0.13 | 0.0360 |
| Brain - Cortex | -0.16 | -2.00 | 0.08 | 0.0450 |
| Adipose - Visceral (Omentum) | 0.17 | 1.90 | 0.09 | 0.0590 |
| Brain - Cerebellum | 0.17 | 1.90 | 0.09 | 0.0620 |
| Skin - Sun Exposed (Lower leg) | 0.13 | 1.70 | 0.08 | 0.0950 |
| Breast - Mammary Tissue | 0.11 | 1.40 | 0.08 | 0.1700 |
| Pituitary | 0.18 | 1.30 | 0.14 | 0.2100 |
| Artery - Aorta | 0.11 | 1.20 | 0.09 | 0.2200 |
| Brain - Hypothalamus | 0.18 | 1.20 | 0.16 | 0.2500 |
| Muscle - Skeletal | 0.07 | 1.10 | 0.06 | 0.2800 |
| Artery - Tibial | 0.08 | 1.00 | 0.07 | 0.3100 |
| Nerve - Tibial | 0.07 | 0.97 | 0.07 | 0.3300 |
| Pancreas | 0.11 | 0.97 | 0.12 | 0.3300 |
| Esophagus - Mucosa | 0.07 | 0.94 | 0.08 | 0.3500 |
| Testis | -0.07 | -0.86 | 0.08 | 0.3900 |
| Heart - Left Ventricle | 0.07 | 0.82 | 0.09 | 0.4100 |
| Ovary | -0.12 | -0.82 | 0.15 | 0.4100 |
| Brain - Hippocampus | 0.09 | 0.81 | 0.11 | 0.4200 |
| Lung | 0.05 | 0.79 | 0.06 | 0.4300 |
| Brain - Nucleus accumbens (basal ganglia) | 0.11 | 0.78 | 0.14 | 0.4400 |
| Brain - Frontal Cortex (BA9) | 0.08 | 0.77 | 0.10 | 0.4500 |
| Liver | -0.08 | -0.64 | 0.12 | 0.5300 |
| Brain - Caudate (basal ganglia) | 0.08 | 0.61 | 0.13 | 0.5400 |
| Artery - Coronary | 0.07 | 0.59 | 0.11 | 0.5600 |
| Heart - Atrial Appendage | -0.06 | -0.58 | 0.10 | 0.5600 |
| Thyroid | -0.04 | -0.51 | 0.08 | 0.6100 |
| Skin - Not Sun Exposed (Suprapubic) | 0.05 | 0.45 | 0.10 | 0.6600 |
| Esophagus - Muscularis | -0.04 | -0.43 | 0.08 | 0.6700 |
| Brain - Anterior cingulate cortex (BA24) | 0.03 | 0.31 | 0.11 | 0.7600 |
| Colon - Sigmoid | 0.02 | 0.22 | 0.10 | 0.8200 |
| Stomach | 0.02 | 0.23 | 0.08 | 0.8200 |
| Vagina | 0.04 | 0.21 | 0.17 | 0.8400 |
| Adrenal Gland | 0.02 | 0.17 | 0.09 | 0.8700 |
| Prostate | 0.02 | 0.13 | 0.13 | 0.9000 |
| Brain - Cerebellar Hemisphere | 0.01 | 0.10 | 0.12 | 0.9200 |
| Small Intestine - Terminal Ileum | -0.01 | -0.05 | 0.10 | 0.9600 |
| Brain - Putamen (basal ganglia) | 0.00 | 0.00 | 0.16 | 1.0000 |
