# Supplementary material for: Genetic polymorphisms of NOS2 and predisposition to fracture non-union: A case control study based on Han Chinese population
Source: PLoS One. 2018 Mar 8;13(3):e0193673. doi: 10.1371/journal.pone.0193673 (PMC5843262; doi:10.1371/journal.pone.0193673)
Supplement: S1 Fig — (DOCX) [file pone.0193673.s005.docx]

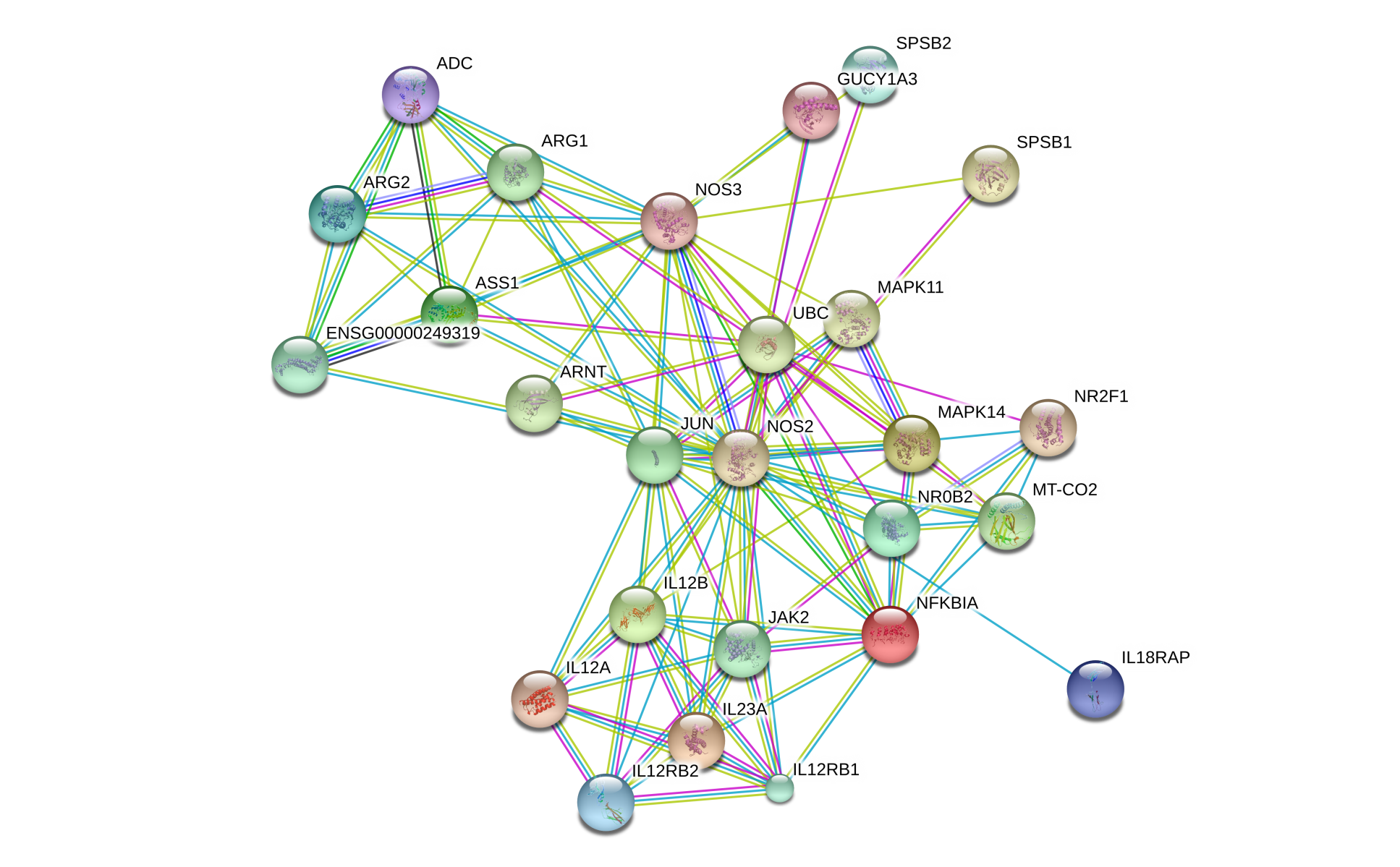


Supplemental Figure S1. Gene network of *NOS2* constructed by Protein-protein interaction (PPI) data.
